# The effect of attitude to risk on decisions made by nurses using computerised decision support software in telephone clinical assessment: an observational study

**DOI:** 10.1186/1472-6947-7-39

**Published:** 2007-11-29

**Authors:** Alicia O'Cathain, James Munro, Iain Armstrong, Catherine O'Donnell, David Heaney

**Affiliations:** 1 Medical Care Research Unit, School of Health and Related Research, University of Sheffield, Regent Court, Sheffield, UK; 2 Audit Scotland, Edinburgh, UK; 3 General Practice & Primary Care, Division of Community-based Sciences, University of Glasgow, Glasgow, UK; 4 Centre for Rural Health, University of Aberdeen, North Inverness, UK

## Abstract

**Background:**

There is variation in the decisions made by telephone assessment nurses using computerised decision support software (CDSS). Variation in nurses' attitudes to risk has been identified as a possible explanatory factor. This study was undertaken to explore the effect of nurses' attitudes to risk on the decisions they make when using CDSS. The setting was NHS 24 which is a nationwide telephone assessment service in Scotland in which nurses assess health problems, mainly on behalf of out-of-hours general practice, and triage calls to self care, a service at a later date, or immediate contact with a service.

**Methods:**

All NHS 24 nurses were asked to complete a questionnaire about their background and attitudes to risk. Routine data on the decisions made by these nurses was obtained for a six month period in 2005. Multilevel modelling was used to measure the effect of nurses' risk attitudes on the proportion of calls they sent to self care rather than to services.

**Results:**

The response rate to the questionnaire was 57% (265/464). 231,112 calls were matched to 211 of these nurses. 16% (36,342/231,112) of calls were sent to self care, varying three fold between the top and bottom deciles of nurses. Fifteen risk attitude variables were tested, including items on attitudes to risk in clinical decision-making. Attitudes to risk varied greatly between nurses, for example 27% (71/262) of nurses strongly agreed that an NHS 24 nurse "must not take any risks with physical illness" while 17% (45/262) disagreed. After case-mix adjustment, there was some evidence that nurses' attitudes to risk affected decisions but this was inconsistent and unconvincing.

**Conclusion:**

Much of the variation in decision-making by nurses using CDSS remained unexplained. There was no convincing evidence that nurses' attitudes to risk affected the decisions made. This may have been due to the limitations of the instrument used to measure risk attitude.

## Background

Variation in decision-making is apparent amongst health professionals in a range of health care settings. In the United Kingdom (UK) there is wide variation in referral to secondary care at general practice and practitioner level [1], with two fold differences between the top and bottom deciles of general practices for hospital admissions, after standardising for population age, sex and deprivation [2,3]. In the United States the percentage of cases classified as urgent in one study varied from 11% to 63% for four nurses, two accident and emergency doctors and two family practitioners [4]. Some of the variation in practice has been explained by differences in case mix. For example socio-demographic patient factors such as social class and the proportion of the population chronically ill explained about half of the variation in hospital admission levels between general practices in the UK [2]. However, attempts to explain the remaining variation by exploring the effect of health professional characteristics on decision-making have resulted in little more of the variation being explained [1,2,5,6]. Investigators of variation in decision-making have recommended moving away from examining the demographic characteristics of health professionals and turning instead to exploring psychological and sociological factors [2], in particular health professional risk-taking in the face of uncertainty [7].

Computerised decision support systems (CDSS) are sometimes used by health professionals with the aim of supporting safety and consistency in their clinical decision-making. In the United Kingdom CDSS is routinely used in NHS Direct in England and Wales, and NHS 24 in Scotland, both of which offer 24-hour national telephone clinical assessment by nurses. One might expect there to be less variation in clinical decision-making between nurses in these services because of the use made of CDSS. However, protocols and computer programmes do not necessarily remove variability in health care [8,9], and in fact we have previously noted that the level of variation in nurses' decision-making in NHS Direct has been similar to that of health professionals in other services [10].

Attempts have been made to explain variation in decision-making when CDSS is used. The type of software, and the length of nursing experience of individuals using the software, explains some of the variation in decision-making [10]. Within the same study, an attempt was also made to explore the decision-making process between nurse and CDSS, and possible influences on it, using qualitative interviews with nurses [10,11]. This helped to explain why variation occurred in decisions made, even when CDSS was used. It was found that, although the CDSS recommends the action which patients should take, nurses can explicitly override this recommendation, or they can influence the recommendation made by the CDSS through the way in which they choose to navigate the system [11]. Within this qualitative study, differences in nurses' attitudes to risk was identified as a possible explanatory factor for variation in the decisions made. Some nurses were very concerned about the risks of under-triaging and missing a serious illness, while others were concerned about over-triaging and overloading a busy service [10]. Nurses viewed the CDSS as a safety net but they also recognised its limitations and the need for nurse expertise. There was variation between nurses in terms of the emphasis they placed on their own professional expertise and intuition, and the expertise embodied within the CDSS. Given the perceived safety net role of the CDSS, a low risk approach for some nurses was to adhere to the software recommendations. Finally, nurses' talk revealed differences in tolerance of uncertainty in their clinical decision-making, which has been identified elsewhere as a possible explanatory factor of variation in decision-making [7].

In light of this, the aims of this study were to assess nurse attitudes to risk in telephone clinical assessment using CDSS, and to examine how far measured differences in attitudes between nurses explained the variation in decisions made. The study was undertaken in NHS 24. As well as offering a 24-hour service, NHS 24 is the frontline service for all out of hours general practice services and therefore a large proportion of calls to it are typically made in the evenings or at weekends. Nurse advisors use CDSS to clinically assess and triage callers and can advise them to self-care, to contact their general practitioner or out-of-hours service immediately or later, or to attend accident and emergency departments urgently or as an emergency via a 999 ambulance. That is, they offer advice on the urgency with which help should be sought, pass that call on if required, or offer advice about the management of a health problem. Typical examples would be a parent of a young child calling to ask whether the child needs to see a doctor immediately, or an adult calling to ask for advice on managing a self-limiting condition. To help nurses develop the skills required for such telephone triage, NHS 24 runs an extensive in-house training programme covering the use of the CDSS system, and the identification and response to potentially high risk clinical situations. In this study the dichotomous outcome variable of whether or not calls were sent to self-care was chosen because advice to 'self-care only' presents both callers and nurses with the clearest risk – that the patient will not be seen by a health professional and may be falsely reassured and their care delayed [10]. Our hypotheses were that nurses who were more concerned about the risk of missing a serious illness, nurses who relied more on the software, and nurses with low tolerance of uncertainty in their clinical decision-making, would be more risk averse and send fewer calls to self care.

## Methods

In April 2005, staff in NHS 24 compiled a list of the 542 nurse advisors employed in the service and sent a two-sided questionnaire to each nurse using the internal postal system. Questionnaires were accompanied by reply paid envelopes and returned directly to the research team. Up to two reminders were sent. Anonymised routine data on all calls undergoing clinical assessment during a six month period in 2005 were obtained from NHS 24, including date of call, time of call, age and sex of patient, triage decision, and identification code of the nurse advisor taking the call. This unique nurse identifier was used to match the triage decision data for an individual nurse to their responses to the questionnaire.

### The questionnaire

A validated tool was required to measure attitudes to risk in nurses undertaking telephone assessment. This tool needed to address clinical decision-making in the face of uncertainty [7], and the other aspects of risk identified in the qualitative study of decision making in nurse telephone assessment [10,11]. Two potential instruments were identified. However, one was not valid for this study because in attempting to measure doctors' tolerance of clinical uncertainty, researchers developed a tool which actually measured doctors' *reactions *to uncertainty, that is the stress related to uncertainty and the reluctance to disclose uncertainty [12]. The other tool – 'the Grol instrument' – had content validity for this study because it measured attitudes to risk-taking in medical decision-making among family practice doctors [13], specifically attitudes towards the risk of missing a serious illness when seeing mostly minor problems. This seemed highly relevant to nurse telephone assessment in NHS 24. We found no validated instruments which measured attitudes to risks specific to telephone assessment so we generated a further 10 items from the qualitative study of clinical decision-making in telephone assessment [10,11].

A questionnaire was constructed which asked questions about the background and work practices of the nurse, as previously asked in a study of NHS Direct [10]. It included questions on length of nursing experience, type of nursing experience prior to joining NHS 24, and length of time worked in NHS 24. Nurses were asked to provide their unique NHS 24 nurse identifier. Additionally, there were 15 statements about attitudes to risk, each with a five point Likert scale ranging from "strongly agree" to "strongly disagree." This section of the questionnaire included the five item 'Grol instrument' on attitudes to risk in clinical decision-making [13], with minor changes made to make it relevant to NHS 24 nurses, and the 10 items generated from our previous work [10,11].

Cognitive testing of the questionnaire was undertaken by asking four NHS Direct nurses to complete the questionnaire in the presence of a researcher and discuss problems with comprehension and reasons why they selected options. This resulted in changes to the instructions, and the options available for some questions. No changes were made to the risk section of the questionnaire but it was noted that nurses found it difficult to decide which option to select for some individual items. They explained that this was due to the complexity of decision-making and the wide range of situations faced by telephone assessment nurses. The questionnaire was then piloted on 25 nurses in an NHS Direct site. Item completion rates, and the spread of data within individual items, were good for the 13 responses received.

### Analysis

First, a descriptive analysis was undertaken of the nurse characteristics. The adapted Grol instrument score was calculated to identify the proportion of nurses with a 'no risk-taking' attitude and to compare that with published data on doctors [13]. Then an exploratory factor analysis was undertaken in SPSS version 12 using the 15 items on risk. The aim of the factor analysis was to identify sets of items which tapped into different aspects of attitudes to risk in telephone triage, such as 'tolerance of uncertainty'. Then STATA version 9 was used to test the explanatory power of nurse-level factors in a multi-level logistic regression. The dependent variable was whether a call was sent to self care or not. The independent variables included in the analysis were age of patient, sex of patient (male, female), time of day of call (in-hours Monday-Friday, out-of-hours evenings and weekends), three variables on nurse background and experience, and the fifteen risk items. Ideally, in comparing the decisions of different nurses, adjustments should be made for case-mix to take account of the type and severity of health problems. However, this information is not routinely available from NHS 24 and therefore case-mix adjustment was only possible using the age and sex of the patient, and the time of the call. In NHS 24 calls are allocated sequentially to nurses on duty and therefore there should be no systematic differences in the case-mix of different nurses except that due to the time of day or night they work. We restricted the analysis to include only nurses who had assessed at least 100 calls during the period examined.

## Results

### Response rate

14% (78/542) of nurses were on maternity leave, long term sick leave, or had left the service, and therefore did not receive the questionnaire. This proportion was similar to that found in a survey of nurses in NHS Direct (94/517 = 18%) [10]. After removing absent nurses, the response rate was 57% (265/464) overall, which was lower than that of studies of NHS Direct, which obtained rates of 70% [10] and 74% [14]. Feedback from the service suggested that substantial organisational change had resulted in a perceived over-surveying of nursing staff and therefore a lower response to this questionnaire. The service was also subject to intense media scrutiny at this time.

### Description of nurses and calls

Descriptive data on nurse characteristics are based on the 265 respondents to the questionnaire and are shown in Table 1. The telephone assessment nurses were mainly female, had over ten years nursing experience, had previously worked in the National Health Service (NHS), and had worked in telephone assessment for over two years. Nurses were also asked how they would describe the main part of their nursing experience and the majority described this as acute hospital experience.

**Table 1:** Characteristics of telephone assessment nurses (N = 265 nurses)

Characteristic	% (n) unless otherwise stated
**Sex**	
Male	10% (27)
Female	90% (238)

**Age**	
Mean (SD)	42 (7.1)

**Length of nursing experience**	
<10 years	9% (24)
10–19 years	41% (108)
20+ years	50% (132)

**Employment 3 months before NHS 24**	
NHS nursing	75% (198)
*Emergency department*	*8% (21)*
*Intensive care*	*14% (37)*
*Other hospital specialty*	*29% (76)*
*Community*	*14% (37)*
*General Practice*	*5% (13)*
*Other*	*5% (14)*

Nursing outside NHS	16% (43)
Working not in nursing	1% (3)
Not employed	2% (5)
Other	6% (16)

**Months worked in NHS 24**	
0–11	18% (48)
12–23	24% (64)
24+	57% (149)

**Main type of experience**	
Hospital	67% (176)
Community	17% (45)
Mixed/Other	16% (43)

Responses to the fifteen risk items are shown in Table 2. Item response was high, with only two items having missing response rates over 2% (items 2 and 4), both part of the adapted Grol instrument. The overall score for the adapted Grol instrument was calculated by taking the mean of the percentages of nurses agreeing or strongly agreeing with each of the five items in the tool [13]. On this measure 59% of nurses had a 'no risk-taking' attitude to clinical decision-making. There was, however, wide variation among nurses' responses to the fifteen risk items (Table 2).

**Table 2:** Responses to 15 risk items (N = 265 nurses)

Items groups by original concepts of risk	Strongly agree	Agree	Neither	Disagree	Strongly Disagree	N = 100%
**Risk tolerance in clinical decision-making**						
1. When in doubt it is preferable to refer to a service than to wait and see	9%	47%	20%	24%	1%	262
2. An NHS 24 nurse must prefer the certain to the uncertain	7%	32%	29%	29%	2%	253
3. An NHS 24 nurse must not take any risks with physical illness	27%	47%	9%	17%	0%	262
4. For physical complaints an NHS 24 nurse should do everything possible to establish the cause of a complaint	16%	46%	12%	23%	2%	258
5. As an NHS 24 nurse you must always be aware that each complaint might be the beginning of a serious illness	13%	50%	14%	20%	3%	262
**Competing expertise of nurse and computer**						
6. I prefer to use a clinical algorithm than to assess calls on my own	6%	27%	26%	38%	4%	263
7. It is best to avoid overriding clinical algorithms unless absolutely necessary	2%	19%	17%	52%	10%	263
8. The most important thing is to follow the agreed algorithms	<1%	8%	22%	56%	14%	262
9. I often feel I know better than the clinical assessment system	3%	22%	40%	31%	3%	260
10. Intuition or gut reaction plays an important role in my clinical decision making	15%	62%	13%	8%	1%	265
11. I have strong views on where particular patients should be advised to go	5%	40%	38%	16%	1%	263
**Competing risks between missing illnesses and overloading services**						
12. It is important not to overload busy services	24%	51%	13%	11%	1%	261
13. To be safe, it is better to send 100 patients to a service unnecessarily than to leave one at home who needs care	1%	12%	19%	57%	11%	263
14. I feel that nurses in NHS 24 tend to send more patients to services than is really necessary	5%	32%	24%	35%	3%	262
**General**						
15. I tend to be quite a cautious person	7%	42%	29%	22%	<1%	261

Factor analysis was undertaken on these fifteen risk items. Two items were excluded due to low correlation with other items. Factor analysis was undertaken on the remaining thirteen items using Varimax rotation and then Promax rotation in case the factors were correlated [15]. The same four factors were identified under both analyses, but the internal reliability of each of these factors was low, with Cronbach's alpha ranging from 0.40 to 0.57 rather than the recommended 0.7 to 0.9 [16]. Also, the adapted Grol instrument did not load on to a single factor and had internal reliability of only 0.50. Combining items into internally reliable factors was not possible and therefore the 15 risk items were tested individually.

We were able to link survey responses to call data for 251 nurses, of whom 211 had taken at least 100 calls in the relevant time period. These 211 nurses had taken a total of 231,112 calls. Calls were mainly triaged outside normal working hours and 16% were triaged to self care (see Table 3).

**Table 3:** Characteristics of calls taken by 211 nurses (N = 231,112)

Characteristic	% (n)
**Sex of patient**	
Male	42% (96090)
Female	58% (135022)

**Age of patient**	
5 years or less	18% (42344)
6 – 15	10% (23812)
16 – 35	24% (55985)
36 – 65	28% (65032)
66 or older	19% (43860)

**Time of call**	
In hours	9% (19832)
Out of hours	91% (211280)

**Triage decision**	
Self care	16% (36342)
Other	84% (194770)

### Effect of nurse background and attitude to risk on decision-making

There was substantial variation in decision-making among the 211 nurses. The proportion of calls sent to self care was 16% overall, but varied three fold between the bottom and top deciles of nurse. Variability was partly explained by the main type of experience of nurses (Table 4). Nurses with a background mainly in the community, for example as practice nurses, district nurses or health visitors, were less likely to send patients to self care than nurses mainly with acute hospital experience.

**Table 4:** Effect of nurse background on proportion of calls sent to self care (211 nurses taking 231,112 calls)

Characteristic	Odds ratio* (95% CI)	p-value
**Length of nursing experience**		0.60
< 10 years	1	
10–19 years	1.04 (0.95, 1.14)	
20+ years	1.14 (1.04, 1.25)	

**Main type of experience**		0.025
Hospital	1	
Community	0.90 (0.86, 0.95)	
Mixed/other	0.96 (0.92, 1.01)	

**Months worked in NHS 24**		0.20
0–11	1	
12–23	1.04 (0.95, 1.14)	
24+	1.08 (0.99, 1.19)	

* odds ratio from multi-level model adjusted for age and sex of patient and time of call

When attitudes to risk were examined as potential explanatory factors, a number of statistically significant associations between nurses' attitudes to risk and their decision to send to self care were evident (Table 5). The odds ratios show the relationship between attitude to risk and the probability of sending patients to self care; the reference group is nurses strongly agreeing with each statement and odds ratios greater than one indicate that nurses were more likely to send to self care. Although many of the odds ratios were in the expected direction, some were not. For example, the hypothesis on risk in clinical decision-making measured by the adapted Grol instrument was that risk averse nurses would be less likely to send callers to self care. Yet this was not consistently the case for these 5 items. Also, there were few examples of smooth gradients of odds ratios from strongly agree to strongly disagree for the 15 items. Smooth gradients were only apparent for three of the four items which mentioned the clinical algorithms of the CDSS.

**Table 5:** Effect of nurse attitudes to risk on proportion of calls sent to self care: odds ratios* based on 211 nurses making 231,112 calls, with numbers of nurses in each category in brackets

Items groups by original concepts of risk	Strongly agree	Agree	Neither	Disagree	Strongly Disagree	p-value
**Risk tolerance in clinical decision-making**						
1. When in doubt it is preferable to refer to a service than to wait and see	1 (22)	1.05 (102)	1.00 (40)	0.86 (44)	- (1)	0.001
2. An NHS 24 nurse must prefer the certain to the uncertain	1 (16)	1.39 (73)	1.22 (55)	1.36 (53)	1.24 (5)	0.001
3. An NHS 24 nurse must not take any risks with physical illness	1 (58)	0.67 (97)	0.95 (21)	1.02 (32)	- (0)	0.06
4. For physical complaints an NHS 24 nurse should do everything possible to establish the cause of a complaint	1 (34)	1.01 (97)	0.63 (23)	1.32 (48)	1.05 (4)	0.002
5. As an NHS 24 nurse you must always be aware that each complaint might be the beginning of a serious illness	1 (31)	0.92 (108)	0.42 (30)	0.81 (37)	1.62 (4)	0.09
						
**Competing expertise of nurse and computer**						
6. I prefer to use a clinical algorithm than to assess calls on my own	1 (13)	1.09 (52)	1.26 (53)	1.69 (81)	1.20 (10)	0.001
7. It is best to avoid overriding clinical algorithms unless absolutely necessary	1 (4)	0.95 (41)	0.99 (35)	1.47 (112)	1.36 (17)	0.001
8. The most important thing is to follow the agreed algorithms	- (0)	1 (15)	0.84 (48)	1.05 (119)	0.97 (27)	0.20
9. I often feel I know better than the clinical assessment system	1 (7)	1.01 (46)	0.96 (89)	0.91 (57)	0.83 (7)	0.002
10. Intuition or gut reaction plays an important role in my clinical decision making	1 (32)	1.09 (132)	0.72 (29)	0.92 (18)	1.00 (2)	0.001
11. I have strong views on where particular patients should be advised to go	1 (11)	1.53 (83)	1.13 (80)	1.41 (34)	1.38 (2)	0.001
						
**Competing risks between missing illnesses and overloading services**						
12. It is important not to overload busy services	1 (53)	1.00 (101)	1.01 (29)	0.98 (24)	1.28 (1)	1.00
13. To be safe, it is better to send 100 patients to a service unnecessarily than to leave one at home who needs care	1 (4)	0.59 (26)	0.52 (37)	0.61 (118)	0.60 (24)	0.11
14. I feel that nurses in NHS 24 tend to send more patients to services than is really necessary	1 (9)	1.36 (66)	0.93 (55)	1.33 (72)	0.99 (7)	0.005
						
**General**						
15. I tend to be quite a cautious person	1 (14)	1.38 (88)	1.48 (63)	1.32 (41)	- (1)	0.001

*adjusted for age and sex of patient and time of call

## Discussion

This study has confirmed that there is variation in decision-making among telephone assessment nurses using CDSS. The attitudes of NHS 24 nurses to risk are comparable to those of some European family doctors; 59% of nurses exhibited a 'no risk-taking' attitude to clinical decision-making, compared with 54% of British doctors, 60% of Belgian doctors and 24% of Dutch doctors [13]. There were a number of statistical associations between nurses' attitudes to risk and the proportion of calls they sent to self care. However, the lack of consistency of relationship within and across items reduces the credibility of these associations. The strong correlations between referral behaviour and attitudes to risk in a study of family doctors using some of the same items were not consistently found here [13]. The only items with any consistency were those which mentioned the computer algorithms. Safety is embodied in the computerised decision-support software [11] and therefore we would expect nurses who felt strongly about the need to use the software to be risk averse and be less likely to use the option of self care. This was the case for three of the four items which mentioned the computer, with some gradient of relationship within items.

Those nurses whose background was mainly in community nursing were less likely to send callers to self care than nurses from a mainly acute hospital background. The effect of type of nursing background on telephone assessment decisions has previously been examined for NHS Direct, and no relationship found, although nurses with longer lengths of nursing experience were more likely to send callers to self care [10]. In our study we found no association between length of nursing experience and clinical assessment, although the odds ratios in our study showed some increase with length of nursing experience. These differences between NHS Direct and NHS 24 are surprising given the similarity of the services and of the research methods used to explore the effect of nurse characteristics on triage decisions. However, one important difference between NHS 24 and NHS Direct is the extent to which each provides out-of-hours services for general practice. Over 90% of NHS 24 calls in this study were out-of-hours (compared with 70% of NHS Direct calls [10]) and in addition NHS 24 takes all general practice out-of-hours calls. Also, the proportion of calls sent to self care in NHS Direct was 40% [10] compared with 16% for NHS 24 in this study. This contextual difference may account for differences between findings for the two services. Indeed the findings may be specific to other contextual issues such as the type of decisions being made. The majority of the decisions here were made in the context of triaging for out of hours general practice services rather than, for example, supporting a patient to make values-sensitive decisions [17].

The development and testing of an instrument to measure attitudes to risk in telephone assessment was not an aim of this study, but was undertaken given the lack of an existing instrument. Although we followed established methods of instrument development, taken together our items did not perform well psychometrically [15]. Our attempts to measure attitudes to risk may, in retrospect, have been insufficiently conceptualised. The risk environment faced by telephone triage nurses is complex, and includes a range of competing risks whose salience may vary from nurse to nurse. For example, the nurse must consider patient safety, patient convenience, management demands for efficiency, the workload of services, and their own professional integrity and reputation. In many situations these risks are in conflict: avoiding one may mean facing another instead. Given such a context, even if a nurse can be said to have an 'aversion to risk', it is unclear how this will impact on their assessment behaviour, since they will be able to choose to avoid only some of the risks in the complex environment they face. The items in our risk measure attempted to address some of the competing risks evident from our previous qualitative work, but may not have been sufficiently closely related to how nurse decision-making actually occurs. We suggest that future quantitative work in this area will benefit from a deeper understanding of the risk environment as perceived by telephone assessment nurses. The use of observation of nurse advisors, or clinical vignettes, to determine nurses' attitudes to risk may be a more useful way forward than the instrument used here.

## Conclusion

Our conclusions are that we found no convincing evidence that nurses' attitudes to risk affected their decisions. However this may have been due to limitations in the way in which we measured attitudes to risk.

## Competing interests

The author(s) declare that they have no competing interests.

## Authors' contributions

JM, COD, and DH conceived the study. JM and AOC designed the study. IA facilitated acquisition of data. AOC analysed the data. All authors contributed to interpretation of data and guided further analysis. AOC drafted the manuscript and all authors made critical contributions to further drafts. All authors read and approved the final manuscript.

## Pre-publication history

The pre-publication history for this paper can be accessed here:


